# Factors Influencing the Use of Walking Aids by Frail Elderly People in Senior Day Care Centers

**DOI:** 10.3390/healthcare11060858

**Published:** 2023-03-14

**Authors:** Yuya Sakano, Shin Murata, Akio Goda, Hideki Nakano

**Affiliations:** 1 Kissho-Home of Social Welfare Corporation Seiwaen, Kyoto 601-8389, Japan; 2 Graduate School of Health Sciences, Kyoto Tachibana University, Kyoto 607-8175, Japan; 3 Faculty of Health Science, Kyoto Tachibana University, Kyoto 607-8175, Japan

**Keywords:** walking aids, senior day care centers, knee extensor strength

## Abstract

Background: Senior day care centers in Japan are the most commonly used insurance service for frail elderly people, and it is important to examine the factors that influence the use of walking aids at these centers. We aimed to investigate the factors affecting the use of walking aids by frail elderly patients in senior daycare centers. Methods: A total of 131 frail elderly people (mean age 82.7 ± 6.4 years) formed our study population. They were divided into two groups: solo walking (*n* = 87) and walking aid (*n* = 44). Individuals with cognitive decline were excluded. All participants were assessed using Functional Independence Measure Motor (FIM-M) sub-scores. Grip strength, knee extension strength, the 10 s Chair Stand test for Frail elderly (Frail CS-10), Single Leg Standing (SLS), and Timed Up and Go (TUG) tests were measured and compared between the two groups. Results: The walking aid group scored significantly lower than the solo walking group for five items: FIM-M, knee extension muscle strength, Frail CS-10, SLS, and TUG (*p* < 0.05). Logistic regression analysis revealed that knee extension muscle strength was the only factor that affected the use or disuse of walking aids (*p* < 0.05). The cut-off value of the knee extensor muscle strength-to-weight ratio for walking alone was 28.5%. Conclusions: Knee extensor muscle strength was the most important factor in determining the use of a walking aid.

## 1. Introduction

Walking is one of the most important activities of daily life. However, walking can be impaired by several factors, including acute and chronic diseases, and physiological changes due to aging [1,2,3]. Walking disruptions in the elderly population adversely affect their participation in social activities and their quality of life [4]. In addition, walking disturbances increase the risk of falls [5]. Falls in elderly people cause serious injuries, such as bone fractures [6], and often result in death [7]. Therefore, it is important to prevent falls [8,9,10].

Elderly people use walking aids to prevent falls. These aids maintain walking ability and improve balance by increasing the base of support and lateral stability. They also reduce the load on the lower limbs, thereby enhancing balance [8]. Studies have reported that elderly people felt more stable and safer when using walking aids [11,12]. Walking aids can help individuals maintain or regain social activity and improve their confidence and sense of security [13]. On the other hand, it has been reported that the use of walking aids was associated with a 2.6-fold (confidence interval—1.2–4.6) increased risk of falling [14], which could be due to the inappropriate use of walking aids [15,16]. Furthermore, since walking aids reduce the load on the lower limbs [17], their use from an early stage may lead to a decline in lower limb function. Therefore, it is important to conduct objective and appropriate evaluations and recommend the use of walking aids to elderly people who need them the most.

Predictions using equipment for fall prevention [18,19] and studies using machine learning algorithms [9] have recently been reported. However, the decision to use a walking aid is often subjective, and the factors influencing the use of a walking aid have not been fully investigated. A previous study examined the factors influencing solo walking in elderly hospitalized patients and reported that knee extension muscle strength was significantly associated with walking ability in these individuals [20]. On the other hand, it has also been reported that walking aids can interfere with an individual’s ability to maintain balance in certain situations [8]. As senior day care centers in Japan are the most commonly used insurance service for frail elderly people [21], it is important to examine the factors that influence whether or not this population uses walking aids. We searched for “senior day care centers” and “walking aids” using various databases, including PubMed, Web of Science, and Scopus; however, as of 20 February 2023, we were unable to identify any such studies. Therefore, the purpose of this study was to evaluate gait, balance ability, and muscle strength in frail elderly patients in a senior day care center, and to identify factors that influenced their use of walking aids. The hypothesis of this study was that individuals who use walking aids may have decreased knee extension muscle strength, balance function, or both.

## 2. Materials and Methods

### 2.1. Study Design

This cross-sectional observational study was conducted at a senior day care center in Kyoto, Japan, in 2021. Posters were displayed in the day care center for the elderly for one month to recruit participants. This study was conducted in accordance with the tenets of the 1975 Declaration of Helsinki (revised in 2013). Participants were fully informed about the purpose and content of the study, benefits and risks, protection of personal information, and refusal or withdrawal of consent. All individuals participated voluntarily. This study was conducted with the approval of the Research Ethics Committee of Kyoto Tachibana University (Approval No. 21-24).

### 2.2. Study Participants

**Figure 1:**
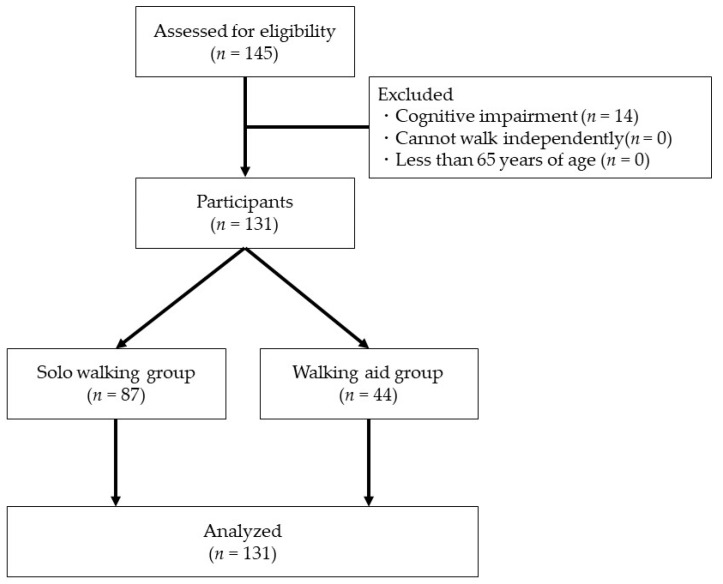
Participant selection flowchart.

A total of 145 elderly people of either sex from a senior day care center formed our study population. The inclusion criteria were: (1) subjects who used long-term care insurance services in Japan [22,23], (2) elderly persons aged 65 or older, and (3) subjects who walked (with or without a walking aid, such as a cane) as a means of daily transportation. Exclusion criteria were: (1) those who had difficulty walking independently, (2) those who were 64 years old or younger, and (3) those who had cognitive impairment. Cognitive impairment was defined as a Mini Mental State Examination (MMSE) score of less than 20 points [24]. This study was conducted on frail elderly patients using a senior day care center, and no exclusions were made based on disease. The nurse checked all vital signs before taking measurements, and only those who were allowed to do so participated. After excluding those who met the exclusion criteria, 131 subjects were included in the final analysis (Figure 1). Sample size was calculated using G * Power [25]. G * Power was set as follows: test family: t-test without correspondence; effect size: 0.5; alpha error probability: 0.05; and power (1-beta error probability): 0.80. The total sample size was calculated to be 128.

The study participants were divided into two groups: those who did not use walking aids when they walked (solo walking group) and those who used walking aids (walking aid group). 

### 2.3. Measurements

First, the MMSE was administered to all participants to determine declines in cognitive function. Next, physical function was assessed in those without cognitive decline. Physical function assessment included 13 items of the Functional Independence Measure (FIM) related to locomotion (FIM motor sub-scores: FIM-M), grip strength, knee extension muscle strength, 10 s Chair Stand test for Frail elderly (Frail CS-10), Single-Leg Standing (SLS), and Timed Up and Go (TUG) tests. Measurements were performed by a physical therapist and an occupational therapist with more than 10 years of clinical experience. Participants’ names were converted to ID numbers by the occupational therapist, who was blinded to the process. The MMSE is widely used to evaluate general cognitive function [26]. The scores range from 0 to 30, and a score of less than 20 indicates cognitive impairment [24].

The FIM-M measures the amount of care for Activities of Daily Living (ADL) [27]. The FIM-M consists of 13 items: six self-care items (score range: 6–42 points), two toileting items (score range: 2–14 points), three transferring items (score range: 3–21 points), and two moving items (score range: 2–14 points). The total score ranges from 13 to 91, with higher scores indicating more independent ADLs.

Grip strength was measured using a digital grip strength meter T2177 (Toei Light Co., Saitama, Japan), according to the New Physical Fitness Test method [28]. Participants were instructed to stand with their feet shoulder-width apart and arms hanging naturally and to grip the digital grip strength meter with maximum force. Measurements were performed twice on both sides, and the average of the maximum values of both sides was used as the measured value. The average grip strength of elderly Japanese subjects (67.0–79.8 years old) who were not frail was reported to be 33.1 kg for men and 20.9 kg for women [29].

Knee extension muscle strength was measured using the hand-held dynamometer (HHD) Mobie MT-100 and MT-150 (Sakai Med Co., Ltd., Tokyo, Japan). The participants were seated in an end-sitting position, and the length of the HHD belt was adjusted and fixed such that the knee joint was in 90° flexion. The participants were then instructed to extend the knee joint to the maximum extent possible. Measurements were performed twice on each side, and the average of the maximum values on the left and right sides was taken as the measured value and converted to a percentage of body weight [30]. The incidence was reported to be 28.1 ± 9.1% among frail elderly Japanese individuals [31].

Frail CS-10 is a testing method that has been associated with Activities of Daily Living (ADL) in frail elderly people [32]. The participants were seated in a chair without armrests and instructed to stand and sit repeatedly after a start signal. The number of repetitions was measured for 10 s, counting from the sitting to standing position with full extension of the knee joint and back to sitting again as one repetition. Measurements were performed twice, and the higher count was taken as the measured value. Frail elderly Japanese individuals performing Frail CS-10 were able to stand an average of 3.4 times [32].

The SLS test was performed with slight modifications of the methods used in previous studies [33]. A digital stopwatch was used for the measurements. The participants were instructed to raise one leg forward from a standing position, with both hands on their hips. Time was measured from the moment the foot was raised off the ground to the moment when either the foot touched the ground again or the upper limb left the waist. The average of maximum values on the left and right sides was used as the measurement value. 

TUG was measured as the time elapsed from a sitting position in a 40 cm high chair without armrests to standing up, walking around a pylon 3 m away, and sitting back down. Individuals using walking aids were instructed to use their usual aid and walk as fast as possible. Measurements were performed twice, and the shortest time was taken as the measured value [34]. The average TUG of non-frail elderly Japanese is reported to be 8.9 s [35].

### 2.4. Statistical Analysis

SPSS Statistics (version 22.0; SPSS Inc., Chicago, IL, USA) was used for statistical analyses, and the significance level was set at *p* = 0.05. The statistical analysis was performed by two physical therapists: one who was involved (PT, MS) in the measurement and one (PT, PhD) who was not. The participants were blinded by the occupational therapist who performed the measurements, and the two physical therapists who performed the analysis were unaware of this information. Basic attributes of the two groups were compared using the Student’s *t*-test for age and MMSE, and the chi-squared test for sex and main disease. Main disease was defined as the disease identified by the participant’s treating physician as the most significant factor leading to frailty. Diseases that affected fewer than five participants were classified as ”other”.

Initially, normality of the FIM-M, grip strength, knee extension strength, Frail CS-10, SLS, and TUG tests was confirmed using the Shapiro–Wilk test. To identify significant differences between the two groups, Mann–Whitney U-tests were performed for items that were not normally distributed. For normally distributed items, an F-test was performed to confirm equal variances, a Welch’s *t*-test was used if the *p*-value was less than 5%, and a Student’s *t*-test was used if the *p*-value was 5% or greater. A χ^2^ test was used for sex-based comparisons.

In addition, logistic regression analysis was used to examine the factors influencing the use of walking aids. In the logistic regression analysis, use or disuse of walking aids was utilized as the dependent variable, and all items that showed significant differences between the two groups were forced as independent variables.

Receiver Operating Characteristic (ROC) curves were created for factors that were significant in the logistic regression analysis. The cut-off values for the ability of participants to walk solo were determined. Area Under the Curve (AUC), bounded by the ROC curve, was calculated. To examine the clinical relevance of the ROC curve, we determined the fit of the regression model using AUC. We also calculated the sensitivity and specificity and adopted the point with the largest Youden index [36] as the cut-off value. 

## 3. Results

Of the 145 subjects included in the study, 14 had MMSE scores of less than 20 points; therefore, a total of 131 subjects (male *n* = 49; female *n* = 82; mean age 82.7 ± 6.4 years; mean MMSE score 24.3 ± 2.5) were included in the analysis. These 131 subjects were divided into two groups: the solo walking group (*n* = 87; male *n* = 33; female *n* = 54) and the walking aid group (*n* = 44; male *n* = 16; female *n* = 28). There were no significant differences in the basic attributes between the two groups (*p* < 0.05). Sex, age, MMSE, and main disease were not significantly different between the two groups (*p* < 0.05) (Table 1).

The FIM-M, knee extension strength, Frail CS-10, and SLS values of the walking aid group were significantly lower than those of the solo walking group (*p* < 0.01). However, the TUG time was significantly longer (*p* < 0.01) in the walking aid group. No significant differences were found between the two groups in the context of grip strength and MMSE scores (Table 2). 

**Figure 2:**
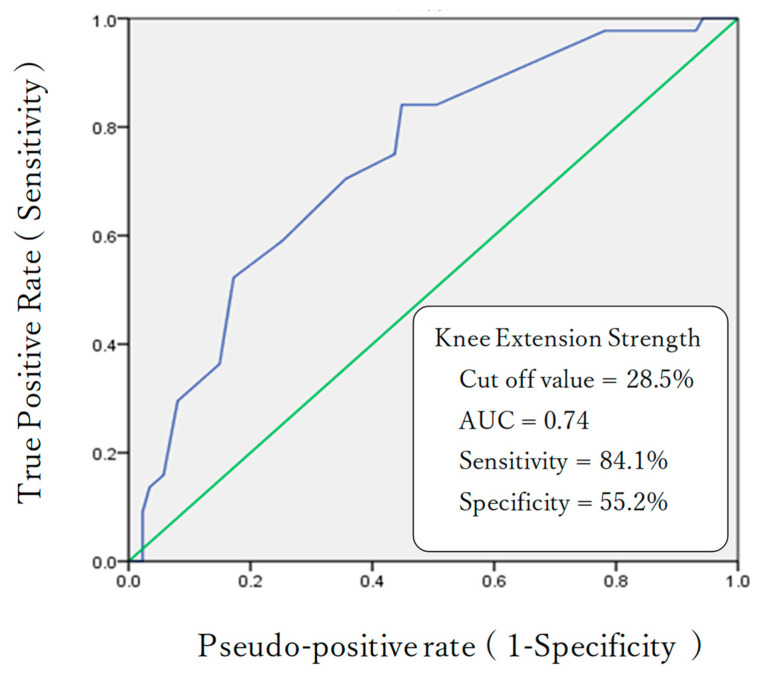
Receiver Operating Characteristic curve for delineating knee extension strength for the use of walking aids. AUC: Area Under the Curve.

A logistic regression analysis revealed that knee extensor muscle strength was the only factor influencing the use of walking aids (Table 3). The cut-off value for knee extensor muscle strength from the ROC curve was calculated to be 28.5% of the body weight. AUC, sensitivity, and specificity were 0.74%, 84.1%, and 55.2%, respectively (Figure 2).

## 4. Discussion

This study aimed to determine the factors that influence the use of walking aids in frail elderly patients at a senior day care center. Our results showed that the walking aid group scored significantly lower than the solo walking group for five items: FIM-M, knee extension muscle strength, Frail CS-10, SLS, and TUG. Multivariate analysis revealed that knee extension muscle strength was the only factor significantly associated with the use of walking aids. The cut-off value for knee extension muscle strength was 28.5%.

Previous studies have reported that low FIM-M scores were associated with falls [27,37]. Knee extension strength [38] and Frail CS-10 are methods used to evaluate lower limb muscle strength. Low values of knee extension strength and Frail CS-10 have reportedly been associated with a high risk of falling. SLS and TUG are balance assessment methods. SLS has been reported to increase the risk of falls at lower values [33]. TUG has been reported to increase the risk of falls at higher values [39]. Thus, it is clear that the significant differences observed between the two groups with regard to these five items increased the risk of falling. Individuals in the walking aid group in this study were at risk of falling and used walking aids to prevent falls. When walking aids were used to avoid falls [40], the subjects in question had significantly lower scores on the five measures related to fall risk compared with those who did not.

Logistic regression analysis was performed to determine whether or not a walking aid had to be used. For this analysis, use of walking aids was considered the dependent variable and the five measures that differed between the two groups as independent variables. Knee extension muscle strength was identified as an influencing factor for the use of walking aids. Walking aids have been reported to be effective in compensating for declines in balance [8]. However, SLS and TUG, which showed significant differences in the univariate analysis, did not show such differences in the multivariate analysis. Although there were significant differences in FIM-M, knee extension muscle strength, Frail CS-10, SLS, and TUG between the two groups, each of these measures alone was not a determinant of whether a walking aid was to be used. As balance function correlates with knee extension muscle strength in the elderly [41], we speculated that the walking aid group had impaired balance function due to diminished knee extension muscle strength and used walking aids to compensate for compromised balance. In addition, previous studies have shown that decreased knee extension muscle strength affected walking stability [42,43]. Multivariate analysis of factors affecting solo walking in elderly individuals reported knee extension muscle strength as an influencing factor [20]. The use of walking aids is recommended on observation of knee extension muscle weakness [8]. However, the load on the lower limbs decreases with the use of walking aids and the amount of load on the walking aid increases [44]. This suggests that the walking aid group may have become unstable and dependent on walking aids, owing to decreased knee extension muscle strength.

We created an ROC curve for knee extension muscle strength and examined the cut-off value to determine whether walking aids were to be used. The cut-off value for knee extension muscle strength was 28.5% (AUC 0.74; sensitivity 84.1%; specificity 55.2%). As the AUC was 0.74, it was considered to have moderate predictive ability [45]. Previous studies have reported that lower-limb muscle strength of at least 28.0% of body weight was required to move stably indoors without the use of walking aids [20]. Our results supported this notion. The sensitivity of the cut-off value was high, and the result was considered valid. However, its specificity was low (55.2%). This may be due to the fact that the mean knee extension strength of the solo walking group was 29.2%, which was close to the cut-off value of 28.5% for knee extension strength. Although the solo walking group had decreased knee extension muscle strength, several of the participants in this group had relatively high stability in walking and had minimum reliance on walking aids. This may be one possible explanation for the similarity between the cut-off value and mean knee extension muscle strength of the solo walking group.

Knee extensor muscle strength declines with age and is a factor in falls [43,46,47]. Walking aids can compensate for muscle weakness and stabilize gait [48]. Therefore, walking aids are expected to be effective in preventing falls in elderly people with reduced knee extension muscle strength. On the other hand, walking aids have the disadvantage of reducing the load on the lower limbs owing to their unloading effect, which leads to a decrease in knee extension muscle strength [49,50]. It has been reported that frail elderly persons can improve their lower limb muscle strength by performing resistance exercises [51,52]. Group training has also been reported to reduce the risk of falling [53]. Therefore, although muscle weakness caused by aging increases the risk of falling, the use of walking aids combined with training can reduce this risk.

This study was conducted to identify factors that influence the use of walking aids and found that knee extension muscle strength was the most important factor. The cut-off value of knee extensor muscle strength for determining whether or not a walking aid is used is 28.5%, suggesting that knee extensor muscle strength is the most important factor in determining whether or not a walking aid is used. This suggests that the use of walking aids, along with resistance exercises and group training, may be effective in preventing falls in frail elderly people with knee extension muscle strength of less than 28.5%, but this study did not go as far as to make that determination. It is important to prevent muscle weakness by continuing to apply weight to the lower limbs without using a walking aid, for those with knee extension muscle strength of 28.5% or greater. Recently, studies have been conducted to predict falls by using instruments, but they require specialized equipment [9,54]. In this study, a hand-held dynamometer was used, which is relatively inexpensive and easy to use [55]. It is clinically significant that we were able to demonstrate the possibility of easily determining the need for the use of a walking aid was used by frail elderly people with the use of a hand-held dynamometer.

This study has several key limitations. First, participants were divided into two groups according to whether they used a walking aid or not, but there was a difference in the number of subjects in the two groups. The reason for this difference is that the participants were not recruited according to whether or not they used a walking aid, but were later divided into the groups. The absence of differences between the two groups was confirmed using a chi-squared test and Student’s *t*-test. It would be preferable to have groups of equal sizes for greater homogeneity. Second, only the use of walking aids was assessed. In addition, this study was conducted as a cross-sectional study, and only the presence or absence of walking aids was determined. Therefore, the study was not able to determine the stability of the effects of walking aids.

## 5. Conclusions

This study revealed that knee extension muscle strength is more important than balance functions such as SLS or TUG in determining the use of walking aids by frail elderly patients at a senior day care center. The cut-off value of knee extension muscle strength for determining the use of walking aids was 28.5%.

## Figures and Tables

**Table 1:** Comparison of basic attributes between the two groups.

	All			Solo Walking Group			Walking Aid Group			*p*-Value
Sex										
Male (n)	49			33			16			0.86
Female (n)	82			54			28			
	Mean	±	SD	Mean	±	SD	Mean	±	SD	
Age (years)	82.7	±	6.4	82.6	±	6.1	82.9	±	6.7	0.80
MMSE (points)	24.3	±	2.5	24.5	±	2.5	24.1	±	2.3	0.42
Main disease										
Bone fracture (n)	37			22			15			0.66
Osteoarthritis (n)	25			16			9			
Cerebrovascular disease (n)	20			13			7			
Aging (n)	15			10			5			
Other (n)	34			26			8			

SD: standard deviation; MMSE: Mini Mental State Examination. Sex, main disease: chi-squared test; age, MMSE: Student’s *t*-test.

**Table 2:** Comparison of measurement results between the two groups.

	**Solo Walking Group**			**Walking Aid Group**			***p*-Value**
	**Mean**	**±**	**SD**	**Mean**	**±**	**SD**	
FIM-M (points)	76.9	±	7.3	73.7	±	6.1	0.00 *
Grip strength (kg)	15.7	±	4.6	14.8	±	4.4	0.21
Knee extension strength (%)	29.2	±	5.4	25.0	±	4.0	0.00 *
Frail CS-10 (times)	3.4	±	1.3	2.6	±	1.3	0.00 *
SLS (s)	2.9	±	3.7	1.5	±	1.1	0.00 *
TUG (s)	14.2	±	4.2	17.4	±	4.0	0.00 *
MMSE (points)	24.5	±	2.5	24.1	±	2.3	0.48

SD: standard deviation; FIM-M: Functional Independence Measure Motor sub-scores; Frail CS-10: 10 s Chair Stand test for Frail elderly; SLS: Single-Leg Standing; TUG: Timed Up and Go test; MMSE: Mini Mental State Examination. FIM-M, grip strength, Frail CS-10, TUG, MMSE: Student’s *t*-test; knee extension strength, SLS: Welch’s *t*-test; * *p* < 0.05.

**Table 3:** Results of logistic regression analysis.

	B	SD	Wald	*p*-Value	Exp (B)	95% CI
FIM-M	0.03	0.04	0.40	0.53	1.03	0.95–1.11
Knee extension strength	−0.14	0.06	6.25	0.00 *	0.87	0.78–0.97
Frail CS-10	−0.10	0.21	0.22	0.64	0.91	0.61–1.36
SLS	−0.27	0.18	2.26	0.13	0.76	0.54–1.09
TUG	0.05	0.07	0.69	0.40	1.06	0.93–1.20
Constant	1.11	3.58	0.10	0.76	3.03	

FIM-M: Functional Independence Measure Motor sub-scores; Frail CS-10: 10 s Chair Stand test for Frail elderly; SLS: Single-Leg Standing; TUG: Timed Up and Go test; B: unstandardized coefficient; SD: standard deviation; CI: Confidence interval; * *p* < 0.05.

## Data Availability

The data used to support the findings of this study are available from the corresponding author upon request. The data are not publicly available because they contain information that can compromise the privacy of the research participants.
